# Kinase Inhibitors of DNA-PK, ATM and ATR in Combination with Ionizing Radiation Can Increase Tumor Cell Death in HNSCC Cells While Sparing Normal Tissue Cells

**DOI:** 10.3390/genes12060925

**Published:** 2021-06-17

**Authors:** Eva-Maria Faulhaber, Tina Jost, Julia Symank, Julian Scheper, Felix Bürkel, Rainer Fietkau, Markus Hecht, Luitpold V. Distel

**Affiliations:** 1 Department of Radiation Oncology, University Hospital Erlangen, Friedrich-Alexander-Universität Erlangen-Nürnberg, 91054 Erlangen, Germany; eva.faulhaber@online.de (E.-M.F.); tina.jost@uk-erlangen.de (T.J.); julia.symank@arcor.de (J.S.); julian.scheper@googlemail.com (J.S.); f.buerkel@gmail.com (F.B.); rainer.fietkau@uk-erlangen.de (R.F.); markus.hecht@uk-erlangen.de (M.H.); 2 Comprehensive Cancer Center Erlangen-EMN (CCC ER-EMN), 91054 Erlangen, Germany

**Keywords:** kinase inhibitors, radiotherapy, targeted therapy, DDR, radiosensitivity, ATM, ATR, DNA-PK

## Abstract

(1) Kinase inhibitors (KI) targeting components of the DNA damage repair pathway are a promising new type of drug. Combining them with ionizing radiation therapy (IR), which is commonly used for treatment of head and neck tumors, could improve tumor control, but could also increase negative side effects on surrounding normal tissue. (2) The effect of KI of the DDR (ATMi: AZD0156; ATRi: VE-822, dual DNA-PKi/mTORi: CC-115) in combination with IR on HPV-positive and HPV-negative HNSCC and healthy skin cells was analyzed. Cell death and cell cycle arrest were determined using flow cytometry. Additionally, clonogenic survival and migration were analyzed. (3) Studied HNSCC cell lines reacted differently to DDRi. An increase in cell death for all of the malignant cells could be observed when combining IR and KI. Healthy fibroblasts were not affected by simultaneous treatment. Migration was partially impaired. Influence on the cell cycle varied between the cell lines and inhibitors; (4) In conclusion, a combination of DDRi with IR could be feasible for patients with HNSCC. Side effects on healthy cells are expected to be limited to normal radiation-induced response. Formation of metastases could be decreased because cell migration is impaired partially. The treatment outcome for HPV-negative tumors tends to be improved by combined treatment.

## 1. Introduction

With the rise in new kinase inhibitors (KI) being approved by the FDA in recent years, this form of targeted therapy has become an increasingly important modality in cancer therapy [1]. A possible target for these KI is the DNA damage-response system (DDR) [2]. The DDR helps repair DNA double-strand breaks (DSB) mainly through two different pathways: homologous recombination (HR) and non-homologous end-joining (NHEJ), with HR being more precise and complicated and NHEJ being quicker but error-prone [3,4]. Tumors are often HR deficient and must rely on NHEJ to repair damage inflicted to their DNA [5]. This can be therapeutically exploited with KI of the DDR. They can inhibit the remaining repair pathways of the malignant cells and, by leaving them no more options to correct DNA damage, induce, e.g., cell death [3].

This method of action prompts a combination of KI with a DNA damaging agent. Chemotherapeutics could be used for that as well as ionizing radiation (IR) [6,7]. IR induces DSBs, which can accumulate in tumor cells and consequently lead to cell death [8]. It is a common treatment modality on its own, as well as in combination with other forms of therapy [9,10]. An example for concomitant treatment with KI is the treatment of metastatic melanoma, where the BRAF inhibitor vemurafenib is applied alongside radiotherapy [11].

In the current work, we focused on head and neck squamous cancer cell carcinoma (HNSCC). Tumors of the head and neck represent the sixth most common type of cancer in the world [10,12]. Common treatment modalities are IR of radiochemotherapy (RCT) as well as surgery and chemotherapy, depending, for example, on tumor location and stage [10,12]. Despite these options, the prognosis is still often poor [13]. Two main entities of HNSCC are HPV-positive and HPV-negative tumors, which have a different genetic backgrounds and are known to react differently to ionizing radiation. HNSCC tumors caused by HPV appear especially in the oropharynx and are more sensitive to IR than tumors where risk factors such as tobacco and alcohol play the main role in tumor genesis [14,15]. HPV status is therefore a factor to be considered in the treatment regime for HNSCC tumors.

It is known that the phosphatidylinositol 3-kinase-related kinase (PIKK) pathways of the DDR are important for HNSCC cells and that DDR inhibitors have shown promising results in trials when combined with KI alongside IR [3,16,17]. The PIKK family contains, among others, ataxia telangiectasia mutated (ATM), ataxia telangiectasia and Rad3-related protein (ATR), DNA-dependent protein-kinase (DNA-PK), and mammalian target of rapamycin (mTOR) [3,18]. Multiple inhibitors of each of the kinases are currently being investigated in clinical trials, such as CC-115, a dual inhibitor of both DNA-PK and mTOR (NCT02833883, NCT01353625), ATM-inhibitor AZD0156 (NCT02588105), and ATR-inhibitor VE-822 (synonyms: berzosertib, VX-970, M6620) (NCT02487095, NCT02589522), [3]. Favorable effects for a combination of IR with CC-115 have already been described in melanoma cells, where an increased radiosensitivity was observed in malignant cells [19]. Additionally, KI targeting DNA single-strand break repair pathways such as PARP1 and PARP2 showed promising results when PARP inhibitors were combined with irradiation [20].

These three KI targeting the DDR are the subject of our research, where we combined these KI with IR to investigate the effectiveness of a concomitant therapy in comparison with sole IR in HNSCC tumor cells and normal skin fibroblasts. Combining KI of the DNA damage repair with ionizing radiation, which classically leads to DNA damage, is of great interest for us because HNSCC includes partly radioresistant subtypes. However, due to their radiosensitizing effects [21,22], KI also pose a risk for the normal tissue surrounding the tumor, which might be affected by combined treatment with KI and IR. It has been observed that increased side effects can occur when IR and KI are combined [23,24,25,26]. Nevertheless, it is known that tumor cells harbor mutations in their DDR system in contrast to healthy non-malignant cells. It is therefore important to look not only at the tumor cells, but at normal tissue as well. Hence, we included primary skin fibroblasts from healthy donors as a reference in all our experiments. In this study, we hypothesize to enhance radiotherapy for HNSCC with a multimodal approach using DDR inhibitors.

## 2. Materials and Methods

### 2.1. Cell Culture and Inhibitors

Primary human fibroblasts SBLF7 and SBLF9 were derived from the skin of a healthy donor as described previously [27]. In brief, skin biopsies of the cutis and subcutis were taken after local anesthesia. The biopsy was cut in small pieces that were then placed in tissue culture flasks and covered with a drop of medium (40% fetal bovine serum). After the skin pieces had attached to the culture flasks and the first fibroblasts had grown out, they were covered with F-12 Medium (Gibco, Waltham, MA, USA). The tissue samples were obtained after informed consent of the donor. Ethical approval was obtained from the Ethics Committee of the medical faculty of the Friedrich-Alexander-Universität Erlangen-Nürnberg (204_17 BC). The established and commercially available HPV-negative HNSCC (head and neck squamous cell carcinoma) cell lines HSC4 and CAL33 as well as the HPV-positive cells UM SCC 47 and UD SCC 2 were obtained courtesy of Dr. Thorsten Rieckmann from the University of Medical Centre Hamburg-Eppendorf, Germany.

Fibroblasts were cultured using F-12 (Gibco, Waltham, MA, USA), with 15% fetal bovine serum (FBS, Sigma-Aldrich, St. Louis, MO, USA), 5% non-essential amino acids (NEA, Gibco, Waltham, MA, USA) and 1% penicillin-streptomycin (Gibco, Waltham, MA, USA). HNSCC cells were cultured in Dulbeco’s MEM (Gibco, Waltham, MA, USA) with 10% FBS and 1% penicillin/streptomycin. All cells were incubated in a humidified atmosphere at 37 °C and 5% CO^2^.

The DNA-PK/mTOR inhibitor CC 115 (Selleckchem, Houston, TX, USA), the ATM inhibitor AZD0156 (Selleckchem, Houston, TX, USA), and the ATR inhibitor VE 822 (Selleckchem, Houston, TX, USA) were dissolved in dimethyl sulfoxide (DMSO, Roth, Karlsruhe, Germany) and stored at −80 °C. Required amounts were thawed anew for every experiment.

### 2.2. Flow Cytometry Analysis of Apoptosis and Necrosis

Cells were seeded into T25 flasks. The number of cells was calculated to reach a confluence of about 70%. After reaching adequate confluence, the medium was changed to a serum-reduced medium containing only 2% of FBS. Subsequently, the cells were treated with the kinase inhibitors. For the DNA-PK inhibitor CC 115 and the ATM inhibitor AZD0156, concentrations of 0, 0.5, 1 and 2 µM were used; for the ATR inhibitor VE 822 cells were treated with 0, 0.1 and 0.5 µM. After 3 h half of the flasks were irradiated with 2 Gy ionizing radiation (IR) by an ISOVOLT Titan X-ray generator (GE, Ahrensburg, Germany). After 48 h of treatment, the cells, including the supernatant, were harvested and stained with 10 µL of a mixture of equal parts APC Annexin V (BD Biosciences, Franklin Lakes, NJ, USA) and 7 AAD (BD Biosciences, Franklin Lakes, NJ, USA) and incubated on ice for 30 min. Subsequently the staining agent was removed, and the cells were centrifuged and resuspended in cold Ringer’s solution (Fresenius, Bad Homburg, Germany) in preparation for flow cytometry analysis. This was performed with the Cytoflex flow cytometer (Cytoflex, Beckman Coulter, Brea, CA, USA). For data evaluation, the Kaluza analysis software (Beckman Coulter, Brea, CA, USA) was used.

### 2.3. Flow Cytometry Analysis of Cell Cycle

Cells were seeded, treated, and harvested in the same way as those for the cell death analysis. Immediately after harvesting, they were fixed in 10 mL of 70% ethanol and 1 mL of starvation medium (containing 2% FBS) and stored at 5 °C for a minimum of 12 h. Cells were then stained with Hoechst 33258 (Molecular Probes, Eugene, OR, USA) and incubated on ice for 60 min. After removing the staining agent, the cells were centrifuged and resuspended in cold Ringer’s solution, and flow cytometry analysis was performed.

### 2.4. Wound Healing Assay

An appropriate number of cells was seeded in cavities of cell culture plates using standard medium. They were incubated at 37 °C for 24 h to develop a monolayer. After 24 h, the cell culture medium was changed to a starvation medium with 2% FBS. After another 24 h of incubation, the monolayer was scratched once using a 10 µL pipet tip, forming a wound with blunt edges. The supernatant cells were removed carefully, and fresh starvation medium (2% FBS) was added. The remaining cells were treated with the kinase inhibitors CC 115, AZD0156, and VE 822 at the same concentrations that were used for the cell death analysis. Half of the cells were irradiated with a 2 Gy dose. Microscopic images were taken initially (0 h) and after 2, 4, 6, 24, and 48 h. The other half of the cells were immediately returned to the incubator. For the images, a Zeiss Primo Vert and Leica DM IL Fluo microscope were used. The area of the scratch wound was then evaluated using image analyzing software (Biomas, MSAB, Erlangen, Germany). With the size of the scratch at 0 h determined to be 100%, a decrease in size over 48 h was then shown in graphs. Additionally, the area under the curve (AUC) for each graph was calculated to allow a comparison among the various cell lines and KI combinations. This was done by calculating both the AUC between 0 and 24 h and between 24 and 48 h. With the maximum value for each of the time frames being “1”, the highest possible value for the AUC over 48 h was “2”, representing no migration.

### 2.5. Colony Forming Assay

An adequate number of cells was seeded in 6-well plates containing 3 mL of fresh medium. After 24 h, different concentrations of DNA damage repair inhibitors were added. After another 3 h, cells were irradiated with a 2 Gy dose. Medium was exchanged with drug-free medium 24 h after KI treatment. Cells were incubated from 10 up to 14 days and afterwards stained with methylene blue (#66725, Sigma Aldrich, München, Germany) for 30 min. Colonies containing more than 50 cells were counted. Plating efficiency (PE) and survival fraction (SF) were calculated. Survival curves for untreated and treated (CC-115, AZD0156, VE-822) cells were plotted, and an additional survival curve was generated after normalizing for the cytotoxicity induced by CC-115, AZD0156 or VE-822.

### 2.6. Statistical Analysis

For the graphs and statistical analysis, GraphPad Prism 8 software (San Diego, CA, USA) was used. Cell death and cell cycle data were analyzed by an unpaired, two-tailed Mann-Whitney U-test while for the cell migration data an unpaired, one-tailed Mann Whitney U-test was used. *p*-value ≤ 0.05 was determined as significant.

### 2.7. Ethics Approval and Consent to Participate

Ethical approval was obtained in the Department of Dermatology, Universitätsklinikum Erlangen following approval by the institutional review board (Ethik-Kommission der Friedrich-Alexander-Universität Erlangen-Nürnberg, approval No. 204_17 Bc). The donors provided written informed consent.

## 3. Results

We studied the effect of a combined treatment of ionizing radiation and kinase inhibitors on HNSCC cells. The ATM inhibitor AZD0156, the ATR inhibitor VE 822 (synonyms: berzosertib, VX-970, M6620), and the dual DNA PK/mTOR inhibitor CC 115 and the HPV-negative cell lines HSC4 and CAL33 as well as the HPV-positive cell lines UM SCC 47 and UD SCC 2 were used. Because the normal skin tissue above or around the tumor is usually also affected by IR therapy, we also studied the effect of combined therapy on the normal skin fibroblasts SBLF7 and SBLF9.

**Figure 1:**
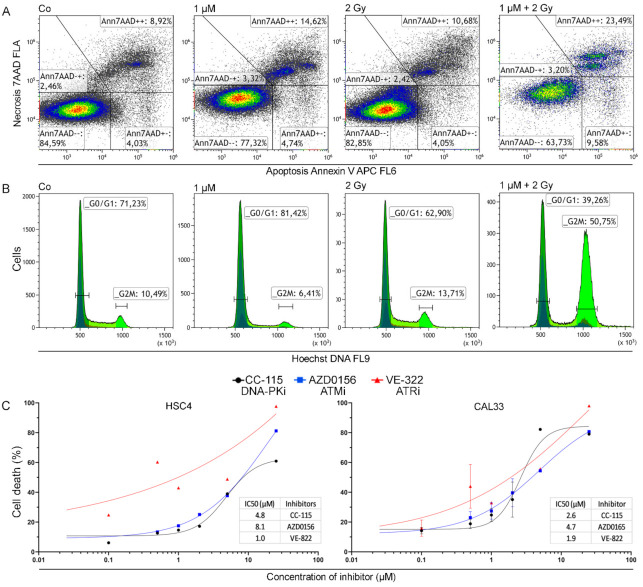
Gating strategies for cell death and cell cycle data and dose escalation studies. (**A**) Dot plot of CAL33 cells untreated, treated with AZD0156, irradiated with 2 Gy, and combined IR and KI treatment to demonstrate the gating strategy of cell death data acquired from flow cytometry analysis. (**B**) Exemplary histograms depicting flow cytometry analysis of cell cycle with focus on G0/G1 and G2/M phases. Histograms show CAL33 cells untreated, treated with AZD0156, irradiated with 2 Gy, and combined IR and KI treatment. (**C**) Dose escalation study for the induction of apoptosis or necrosis (cell death) by the inhibitors CC 115, AZD0156, and VE 822 conducted on HSC4 and CAL33 cells. Each value represents mean ± SD (*n* = 3).

Cell death (apoptosis and necrosis) was evaluated by staining the cells with Annexin V APC and 7 AAD. Annexin V APC positive cells were defined as apoptotic cells, and the Annexin V APC and 7 AAD positive cells were defined as necrotic. Cells that showed no signal for either of the staining agents were classified as viable cells (Figure 1A). Further representative dot plots of cell death analysis are depicted in the supplement (Figures S1–S3).

Furthermore, we investigated cell cycle distribution for all the cell lines and inhibitors using Hoechst 33258 staining and flow cytometry to determine if there was a G2/M phase arrest (Figure 1B). The representative histograms show a pronounced G2/M arrest under treatment with IR and the AZD0156 in the HPV-negative CAL33 cell line. Additional representative histograms of Hoechst (DNA) staining are depicted in the supplement (Figures S4–S6).

Cell death for all three inhibitors was determined by apoptosis and necrosis induction. A dose escalation study was performed using HSC4 and CAL33 cells (Figure 1C) as well as HPV-positive cell line UM-SCC-47 (Figure S7). Associated IC50 values were calculated, and in both cell lines the ATR-inhibitor VE-822 was most effective with an IC50 of 1.0 µM and 1.9 µM. The DNA PK inhibitor CC-115 had a higher IC50 of 4.8 µM and 2.6 µM, and for the ATM inhibitor AZD0156 the IC50 values were highest, with 8.1 µM and 4.7 µM. We used these as a basis for determining sensible doses of the inhibitors for our experiments.

### 3.1. Concomitant KI and IR Treatment Leads to Increase of Cell Death

**Figure 2:**
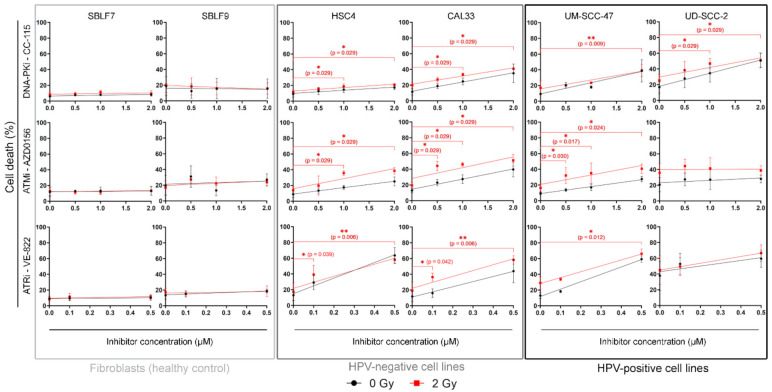
Flow cytometry analysis of cell death. Cells are grouped into normal donor cells (SBLF7, SBLF9), HPV-negative (HSC4, CAL33), and HPV-positive (UM SCC 47, UD SCC 2) HNSCC cell lines. Cell death was determined through flow cytometry by apoptosis (Annexin V) and necrosis (7AAD) detection. Irradiated samples are compared to non-irradiated samples. Effects of increasing concentrations of kinase inhibitors on each cell line are shown. For CC 115 and AZD0156 0.5, 1 and 2 µM, for VE 822 0.1 and 0.5 µM of the inhibitor were used. Each value represents mean ± SD (*n* ≥ 3). Significance was determined by two-tailed Mann Whitney U test * *p* ≤ 0.05 and ** *p* ≤ 0.01.

All cells were treated with 0.5, 1, and 2 µM of CC-115 and AZD0156 and with 0.1 and 0.5 µM of VE 822 (Figure 2). For the primary skin fibroblasts, no significant increase in cell death was observed for the irradiation, the inhibitors, or the combination. In malignant cell lines, cell death increased linearly with inhibitor dose, both with inhibitor treatment alone and with combined treatment. A dual therapy of irradiation and inhibitor is more effective in inducing cell death than IR alone. In part, it depends on the concentration of KI as shown in 10 out of 12 cell line/inhibitor pairs. The dual treatment seemed to have a stronger effect on the HPV-negative cell lines than on the HPV-positive ones. ATM inhibitor AZD0156 proved particularly effective in CAL33 and UM SCC 47 cells.

We also calculated the slope and offset of the linear dose-response relationship to quantify the sensitivity of the cell lines at different concentrations of KI (Table 1).

### 3.2. KI Mainly Arrests Cancer Cells in G2/M

**Figure 3:**
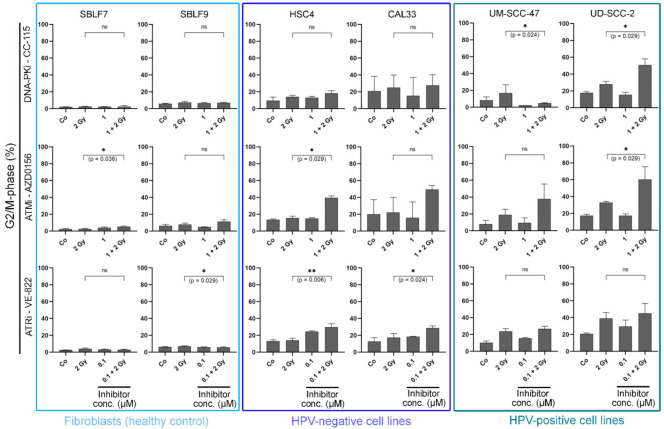
Cell cycle analysis. Cells are grouped into normal donor cells (SBLF7, SBLF9) and HPV-negative (HSC4, CAL33), and HPV-positive (UM SCC 47, UD SCC 2) HNSCC cell lines. The graphs show the proportion of cells in the G2/M phase of the cell cycle and the alteration of G2/M phase under treatment. Cells were either untreated or received IR, KI, or a combination of IR and KI. For CC 115 and AZD0156 1 µM was used, for VE 822 0.1 µM was used. Each value represents mean ± SD (*n* ≥ 3). Significance was determined by two-tailed Mann Whitney U test * *p* ≤ 0.05 and ** *p* ≤ 0.01.

Cells are known to be more sensitive to IR in the G2 phase or during mitosis [28]. We compared the amount of cells in the G2/M phase that only received IR treatment with the cells that were treated with both IR and KI (Figure 3). In the two fibroblast cell lines, the G2/M arrest was only slightly pronounced, and the treatment led to hardly any increase in G2/M arrest. In the two HPV-negative cell lines, arrest occurred mainly after the combined treatment. In the two HPV-positive cell lines, arrest was induced by both irradiation alone and combined treatment. AZD0156 and VE-822 seemed to influence the cell cycle more than CC-115 in both normal and tumorous cell lines. CC 115 effectively arrested only HPV-positive cells in G2/M (*p* = 0.024 for UM-SCC-47 at 1 µM; *p* = 0.029 for UD-SCC-2 at 1 µM). There was no clearly recognizable pattern concerning the cell cycle arrest in G2/M for AZD0156. Clear G2/M arrest occurred in one normal, HPV-positive, and both HPV-negative cell lines (SBLF7: *p* = 0.036 at 1 µM, HSC4: *p* = 0.029 at 1 µM, UD SCC 2: *p* = 0.029 at 1 µM). VE-822 clearly arrested HPV-negative cancer cells and also had a limited effect on normal fibroblasts (*p* = 0.006 for HSC4 at 0.1 µM; *p* = 0.024 for CAL33 at 0.1 µM, *p* = 0.029 for SBLF9 at 0.1 µM).

### 3.3. Combined Treatment Synergistically Reduces HNSCC Cell Survival

**Figure 4:**
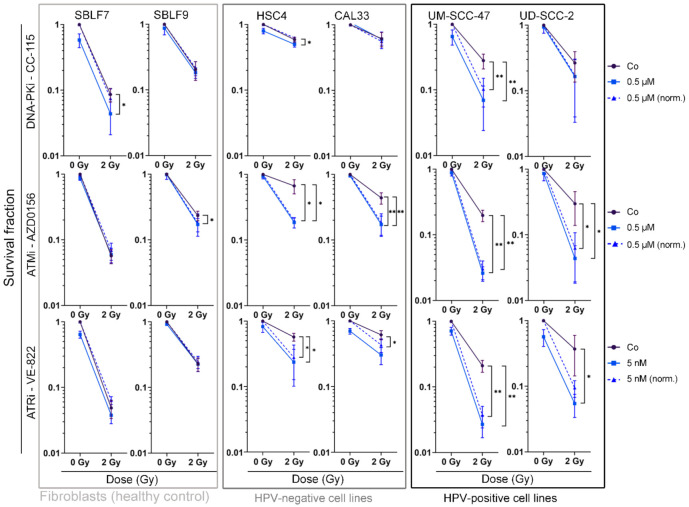
Colony forming assay of HNSCC cell lines and normal controls. Cells are shown grouped into normal cells (SBLF7, SBLF9) and HPV-negative (HSC4, CAL33), and HPV-positive (UM SCC 47, UD SCC 2) HNSCC cells. Graphs show cell survival at 0 Gy and 2 Gy, either with additional treatment of kinase inhibitors (0.5 µM CC 115, 0.5 µM AZD0156, 5 nM VE-822) or without. Additionally, cell survival under KI treatment was normalized to detect synergistic effects. Dashed line represents mean survival fraction normalized to the cytotoxicity induced by KI alone. Each value represents mean ± SD (*n* ≥ 3). Significance was determined by one-tailed Mann Whitney U test * *p* ≤ 0.05 and ** *p* ≤ 0.01. *p* = 0.004: UM SCC 47 + CC 115, UM SCC 47 + CC 115 norm., UM SCC 47 + AZD0156, and UM SCC 47 + AZD0156 norm.; UM SCC 47 + VE 822 and UM SCC 47 + VE 822 norm.; *p* = 0.008: CAL33 + AZD0156 and CAL33 + AZD0156 norm.; *p* = 0.028: CAL33 + VE 822 norm.; *p* = 0.016: SBLF9 + AZD0156; *p* = 0.018: UD SCC 2 + AZD0156 and UD SCC 2 + AZD0156 norm.; *p* = 0.028: UD SCC 2 + VE 822; *p* = 0.050: HSC4 + VE 822, HSC4 + VE 822 norm., SBLF7 + CC-115; *p* = 0.050: HSC4 + CC 115, HSC4 + AZD0156 and HSC4 + AZD0156 norm.

Colony forming assays are considered the gold standard in radiation biology research because they permit estimations about multiple causes of reduced cell viability. We performed these assays in addition to cell death analysis to further investigate the ability of cells to survive and reproduce and to estimate the effect of mechanisms such as senescence and clonogenicity (Figure 4). Representative images of the stained colonies in 6-well format are depicted in the supplement (Figure S8). Possible synergistic effects of combined IR and KI therapy can also be observed here.

Normal skin fibroblasts were clearly affected by IR, showing reduced cell survival, whereas the combination of KI with IR had no significantly increased effect in 4 out of 6 cell line and inhibitor combinations (*p* = 0.029 for SBLF7 + CC 115, *p* = 0.016 for SBLF9 + AZD0156). Compared to IR alone, cell survival was clearly reduced (*p* ≤ 0.050) in all malignant cell lines when treated with AZD0156 or VE 822 in combination with IR. In nine out of 12 pairs of HNSCC cell line and inhibitor, IR and KI therapy showed synergistic effects.

The effect of CC 115 was smaller, with the HPV-negative cell lines HSC4 and CAL33 showing a low response to the inhibitor and the combination therapy. Only in the UM SCC 47 cells did CC-115 have a significant effect on survival (*p* = 0.004).

### 3.4. Migration Behavior of HNSCC Cell Lines Is Strongly Influenced by Inter-Individual Differences

**Figure 5:**
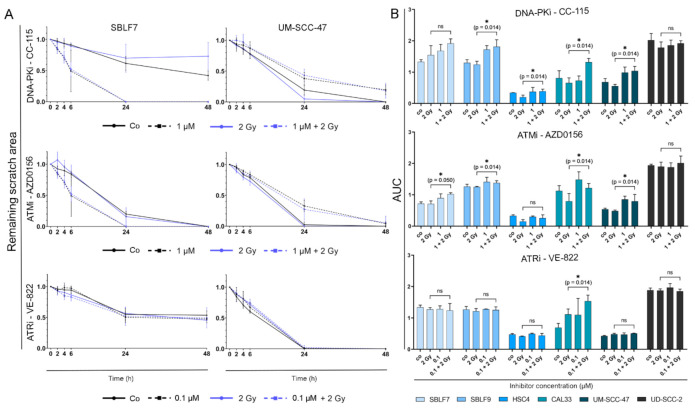
Analysis of cell migration over 24 and 48 h. (**A**) Change of scratch area over 48 h by the example of normal skin fibroblasts SBLF7 and HPV-positive HNSCC cell line UM SCC 47. Graphs show cells untreated, under IR, under KI (1 µM CC 115, 1 µM AZD0156, 0.1 µM VE 822) and under combination therapy. (**B**) Area under the curve (AUC) was calculated representing cell migration over 48 h of four HNSCC cell lines and two normal fibroblast cell lines for all three tested KI. Each value represents mean ± SD (*n* ≥ 3). Significance was determined by one tailed Mann Whitney U test * *p* ≤ 0.05.

We also investigated cell migration under our standard treatment scheme. As an example, migration over time for normal SBLF7 and malignant UM SCC 47 cells is shown (Figure 5A). Representative images of wound healing assays of HPV-negative cell line UM-SCC-47 (Figure S9), one HPV-positive Cal33 (Figure S10), and one healthy cell line SBLF7 (Figure S11) are shown in the supplement.

For the normal skin fibroblasts, treatment with CC-115 resulted in increased cell migration in comparison to untreated or solely irradiated cells. AZD0156 showed an equal tendency. For HPV-positive tumor cell line UM SCC 47, treatment with CC 115 or AZD0156 alone or in combination with IR seemed to slow down cell migration in comparison to untreated or solely irradiated cells. VE 822 did not seem to have an effect on the migration of cells.

The area under the curve (AUC) for a chosen concentration of 1 µM for CC-115 and AZD0156 and 0.1 µM for VE-822 was calculated to determine the extent of cell migration over time (Figure 5B).

Comparing the cells treated with IR alone and the cells receiving combination therapy, it is evident that the addition of CC 115 reduced migration of SBLF9 (*p* = 0.014), HSC4 (*p* = 0.014), CAL33 (*p* = 0.014), and UM SCC 47 (*p* = 0.014) cells. Addition of AZD0156 decreased migration of SBLF7 (*p* = 0.050), SBLF9 (*p* = 0.014), CAL33 (*p* = 0.014), and UM SCC 47 (*p* = 0.014) cells. VE 822 only impaired cell migration of CAL33 cells (*p* = 0.014).

## 4. Discussion

IR therapy is commonly used in HNSCC tumors [10,12]. A combination with modern KI targeting the DNA damage repair network could increase the effectiveness of IR and improve tumor control, which is a highly desirable goal given the currently still poor outcome of HNSCC patients [3,13,16,17]. However, combination therapy also bears the risk of increased unwanted side effects on the surrounding normal tissue [29]. KI from the DNA-DDR pathways have become more and more common in oncology [1]. The inhibitors we chose to investigate all affect kinases in the PIKK pathway of the DDR, with CC-115 being a dual inhibitor of mTOR and DNA-PK, AZD0156 being an inhibitor of ATM, and VE-822 (synonyms: berzosertib, VX-970, M6620) inhibiting ATR [3]. They are not yet approved for the market, but are currently being studied in ongoing phase I-II trials. Tolerability and efficacy of the drugs in combination with chemotherapy has already been proven in patients for VE-822 and CC-115 [7,30].

The focus of our research was on the comparison between effects of sole IR and IR with additional KI treatment. The goal was to determine if a patient who receives IR would be eligible for simultaneous KI treatment. We aimed to assess the effectiveness on tumors and the influence on surrounding cells. Additionally, we analyzed cell cycle and HPV status for better understanding of the intracellular responses and different behavior of the HNSCC cells.

Tumor cells oftentimes have impaired DNA repair mechanisms due to mutations and are therefore more reliant on their remaining repair pathways than normal cells [5]. DNA-PK, ATM, and ATR are involved in these pathways. Tumor cells with deficiencies in these proteins have previously been described as more sensitive to radiation [31]. This suggests that an inhibition of these kinases can increase radiosensitivity, which could result in a better outcome [14]. Our cell death data support this as they show that simultaneous treatment with IR and KI led to higher rates of cell death than IR alone in 10 out of 12 combinations of malignant cell line and inhibitor.

HPV status separates HNSCC tumors into two entities according to tumor genesis [14,15]. HSC4 and CAL33 are HPV-negative cell lines, while UM-SCC-47 and UD SCC-2 are HPV-positive. A combination of KI and IR seemed to induce cell death more effectively in HPV-negative cells than in HPV-positive cells. This is interesting, because HPV-negative HNSCC tumors under current treatment regimens have a worse prognosis than HPV-positive tumors [10,32]. HPV-positive tumors have deficient DSB repair pathways, which leads to increased radiosensitivity compared to HPV-negative cells [14]. If a combination therapy is especially effective in HPV-negative cells, a higher success rate of treatment could be achieved.

When looking at the effect of a combined therapy, it is also of high interest to see if the effects of treatment might be synergistic. This has been described for the combination of other inhibitors with IR [33]. We performed a colony forming assay to be able to assess possible super-additive effects because cell death data from flow cytometry only shows apoptosis and necrosis, while other mechanisms such as senescence might be involved in stalling tumor growth as well [34]. As a natural mechanism of cells to prevent mutation, senescence can be therapeutically exploited. It is a common cellular response to irradiation [35,36]. Notably, inhibition of cell viability through senescence for CAL33 and UM-SCC-47 cell lines and combined therapy with AZD0156 and VE 822 have been previously described, whereas effects of CC-115 on senescence induction could not be observed in this study [37,38]. Our data from the colony forming assay are in line with these findings. They showed no synergistic effects of CC-115 in combination with IR on 3 out of 4 malignant cells lines, whereas we did see an effect of the inhibitor on cell death in flow cytometry. For AZD0156 and VE-822, synergistic effects could be observed for all malignant cell lines. Supra-additive effects on normal skin fibroblasts were not observed for any of the inhibitors.

DNA-PK, ATM, and ATR are all involved in cell cycle regulation [3]. Cells are known to be more sensitive to IR in the G2 phase of the cell cycle, which could be therapeutically exploited by inducing cell cycle arrest in this particular phase [39]. IR itself is capable thereof [40,41], as well as inhibitors of DNA-PK in combination with IR or chemotherapeutic agents [42,43,44]. ATM and ATR inhibition, however, has been described to abrogate G2/M phase block because they appear to be necessary for cell cycle arrest [45,46,47,48]. We investigated whether a combination of KI and IR would lead to an increased number of cells in the G2/M phase by staining cells with Hoechst solution. Although we found that a G2/M block under combined treatment can occur for all inhibitors, there are individual differences between the cell lines. All of them react differently to each of the KI. Overall, inhibition of ATM or ATR appears to have more impact on the cell cycle than inhibition of DNA-PK. However, no clear pattern was recognizable from our experiments, and further investigation is needed to determine the cell line-specific effect of combined treatment on the cell cycle. Because it is well known that ATM harbors a wide range of diverse mutation sites [49], a characterization of the mutation profiles of our cell lines will be part of further projects.

While IR alone can inflict damage to normal cells and cause common side effects such as radiodermatitis or oral mucositis [50,51], it is possible that a concomitant therapy with KI will increase the damage not only on malignant cells, but also on normal ones. Kinase inhibitors have shown radiosensitizing effects on normal cells, causing undesirable side effects such as increased skin reactions when compared to sole IR [23,24]. To determine whether normal tissue could be affected negatively by a concomitant treatment, we investigated the effect of simultaneous treatment on normal skin fibroblasts. Cell death analysis showed us that after two days these cells were not significantly affected by the inhibitor, a one-time irradiation, or a combination of both. The colony forming assay suggested that after 10 days, IR shows an effect on cell survival, but this was not increased by the additional treatment with KI. This could be observed for all three of the investigated KI. These findings indicate that a simultaneous treatment with IR and KI seems to be as safe for tumor-surrounding normal tissue as regular IR treatment.

Another aspect that needs to be considered is distant metastasis. Approximately 15% of patients with locally advanced HNSCC develop distant metastases after definitive RCT [32].

Patients with distant metastasis generally have a poor prognosis and suffer from relevant tumor symptom burden [52,53,54,55]. Cell migration is a requirement for the formation of distant metastases and can be observed with a wound healing assay or scratch assay [55,56,57,58]. We performed such an assay to investigate the influence of KI and IR on the migration of HNSCC cells. The data show that overall, inhibition of ATR in combination with IR did not have a significant impact on cell migration of any cell line except for HPV-negative CAL33 cells, where it slowed down migration. Previously, ATR has been described to promote cell migration, which would lead to the conclusion that inhibition of ATR could decrease metastasis [59]. In combination with Wee-1 kinase inhibition, ATR inhibition has been shown to impair the formation of metastasis [60]. Inhibition of DNA-PK and ATM with simultaneous IR impaired cell migration more extensively. Both inhibitors affected both HPV-positive and HPV-negative cell lines as well as normal tissue cells. Overexpression of ATM has been associated with lymph node metastasis, while inhibition of ATM has been described to reduce cell migration and invasion [61]. Similar findings have been obtained for DNA-PK and its inhibition [62,63]. Interestingly, other factors involved in DNA-DSB repair such as the oncogene PP4R1 and Rad51 are also known for facilitating the formation of metastases when upregulated or overexposed and can be regulated with inhibitors [64,65]. These findings suggest that a combined treatment could have an influence on the formation of metastases through migration, but inter-individual differences between different tumors need to be considered.

## 5. Conclusions

In conclusion, we found that DDRi CC-115, AZD0156, or VE-822 in combination with IR more effectively inhibits tumor growth of HNSCC compared to the single approaches. The combined treatment has a synergistic effect. The combination seems not to affect normal tissue surrounding the tumor more than IR alone, which suggests that no increased side effects should be expected from concomitant treatment. Notably, there were differences in the response between the malignant cell lines, suggesting that the success of treatment will depend greatly on the individual. HPV status might play a role in the success of the treatment, but more in-depth research on that possibility is recommended. Arrest in the G2/M phase of the cell cycle could occur; however, a definite increase in radiosensitivity due to the KI by cell cycle arrest in G2/M could not be observed. Taken together, combined treatment has the potential to be a therapeutic option that could improve tumor control without increasing toxicity.

## Figures and Tables

**Table 1:** Cell death in the form of apoptosis and necrosis induction according to the equation: cell death = m*c + b; where *m* is the slope, *c* is the concentration of the inhibitor, *b* is the offset, and * stands for multiplication.

	Inhibitors					
	CC-115		AZD0156		VE-822	
Cell lines	0 Gy	2 Gy	0 Gy	2 Gy	0 Gy	2 Gy
SBLF7	1.0 * c + 6.4	0.8 * c + 8.7	0.7 * c + 12.1	0.2 * c + 12.2	2.0 * c + 9.2	3.9 * c + 9.7
SBLF9	−0.7 * c + 16.2	−2.4 * c + 19.7	2.0 * c + 21.3	2.9 * c + 19.5	9.7 * c + 13.9	4.1 * c + 16.1
HSC4	3.9 * c + 9.9	4.4 * c + 12.8	8.1 * c + 9.0	12.8 * c + 15.9	98.8 * c + 15.0	76.7 * c + 21.7
CAL33	11.7 * c + 12.3	10.3 * c + 21.3	12.9 * c + 14.4	13.7 * c + 28.3	65.3 * c + 11.0	74.7 * c + 22.2
UM-SCC-47	14.3 * c + 9.3	11.2 * c + 15.8	8.8 * c + 9.1	12.1 * c + 20.6	93.9 * c + 11.8	74.9 * c + 28.3
UD-SCC-2	16.7 * c + 17.9	12.2 * c + 29.7	3.0 * c + 23.2	0.4 * c + 39.8	35.7 * c + 43.0	43.1 * c + 45.1

## Supplementary Materials

The following are available online at https://www.mdpi.com/article/10.3390/genes12060925/s1, Figure S1: Representative dot plots of AnnexinV/7AAD cell death staining of the healthy fibroblasts SBLF7 and SBLF9, Figure S2: Representative dot plots of AnnexinV/7AAD cell death staining of the HPV-positive HSC4 and Cal33, Figure S3: Representative dot plots of AnnexinV/7AAD cell death staining of the HPV-negative UM-SCC-47 and UD-SCC-2, Figure S4: Representative histograms of Hoechst (DNA) cell cycle staining of the healthy fibroblasts SBLF7 and SBLF9, Figure S5: Representative histograms of Hoechst (DNA) cell cycle staining of HPV-positive HSC4 and Cal33, Figure S6: Representative histograms of Hoechst (DNA) cell cycle staining of HPV-negative UM-SCC-47 and UD-SCC-2, Figure S7: Dose escalation study for the induction of apoptosis or necrosis (cell death) by the inhibitors CC 115, AZD0156 and VE 822 conducted on HPV-positive cell line UM-SCC-47, Figure S8: Colony forming assay. Representative images of all tested cell lines (healthy: SBLF7, SBLF9; HPV-neg: HSC4, Cal33; HPV-pos: UM-SCC-47, UD-SCC-2) and inhibitor (AZD0156: ATMi; CC-115: DNA-PKi, VE-822: ATRi), Figure S9: Representative images of wound healing assay of HPV-positive cell line UM-SCC-47 at time points 0 h and 48 h, Figure S10. Representative images of wound healing assay of HPV-negative cell line Cal33 at time points 0 h and 48 h, Figure S11; Representative images of wound healing assay of healthy control cell line SBLF7 at time points 0 h and 48 h.
