# Supplementary material for: Kinase Inhibitors of DNA-PK, ATM and ATR in Combination with Ionizing Radiation Can Increase Tumor Cell Death in HNSCC Cells While Sparing Normal Tissue Cells
Source: Genes (Basel). 2021 Jun 17;12(6):925. doi: 10.3390/genes12060925 (PMC8235750; doi:10.3390/genes12060925)
Supplement: Supplementary file 1 [file genes-12-00925-s001.zip › genes-1265921-supplementary.pdf]

genes

Supplemental Material

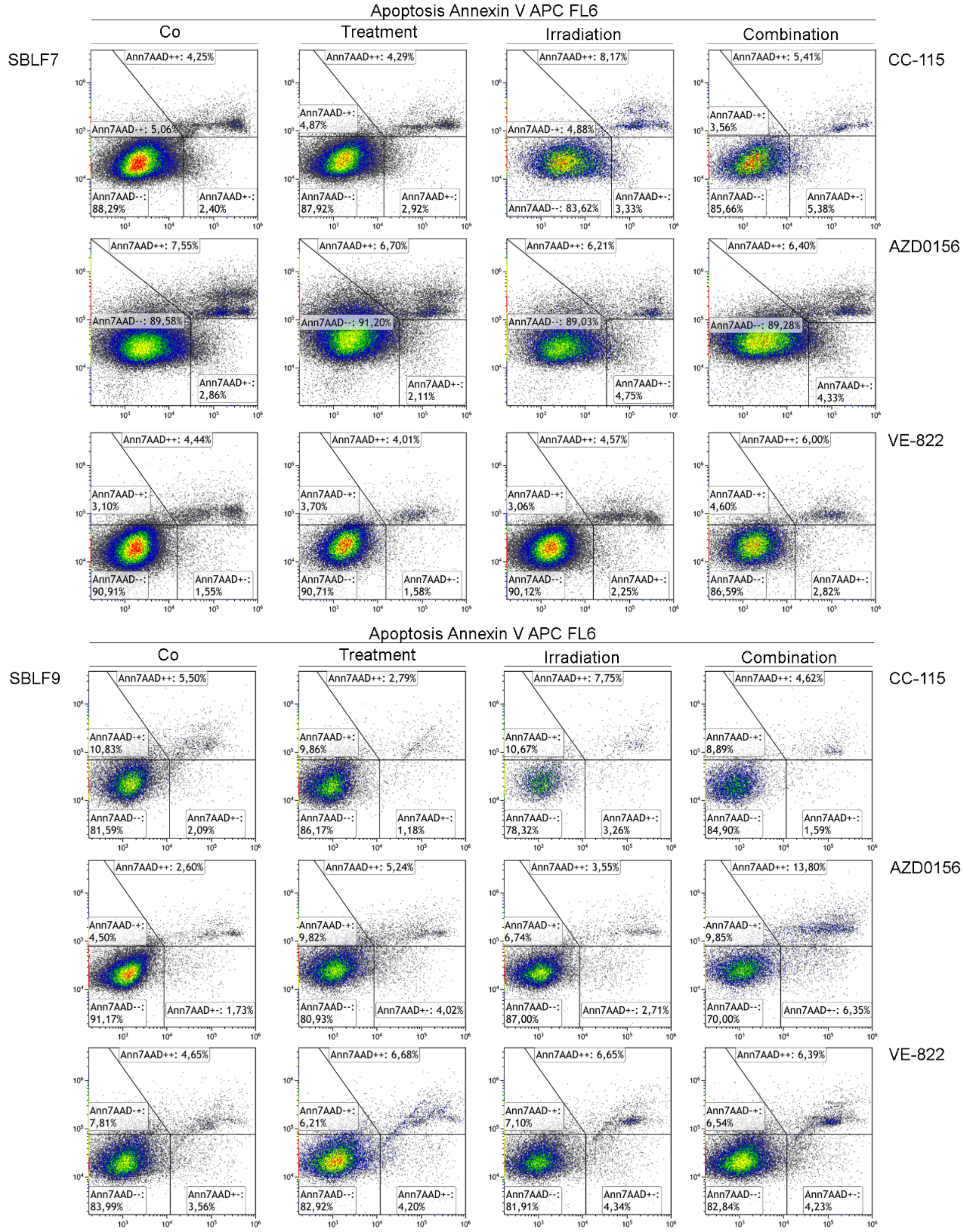

Figure S1. Representative dot plots of AnnexinV/7AAD cell death staining of the healthy fibroblasts SBLF7 and SBLF9. Cells were treated with CC-115 (no inhibitor, 1 µM, 2 Gy irradiation, 1 µM + 2 Gy combination), AZD0156 (no inhibitor, 1 µM, 2 Gy irradiation, 1 µM + 2 Gy combination) or VE-822 (no inhibitor, 0.1 µM, 2 Gy irradiation, 0.1 µM + 2 Gy combination).

GCAT TACG GCAT genes

MDPI

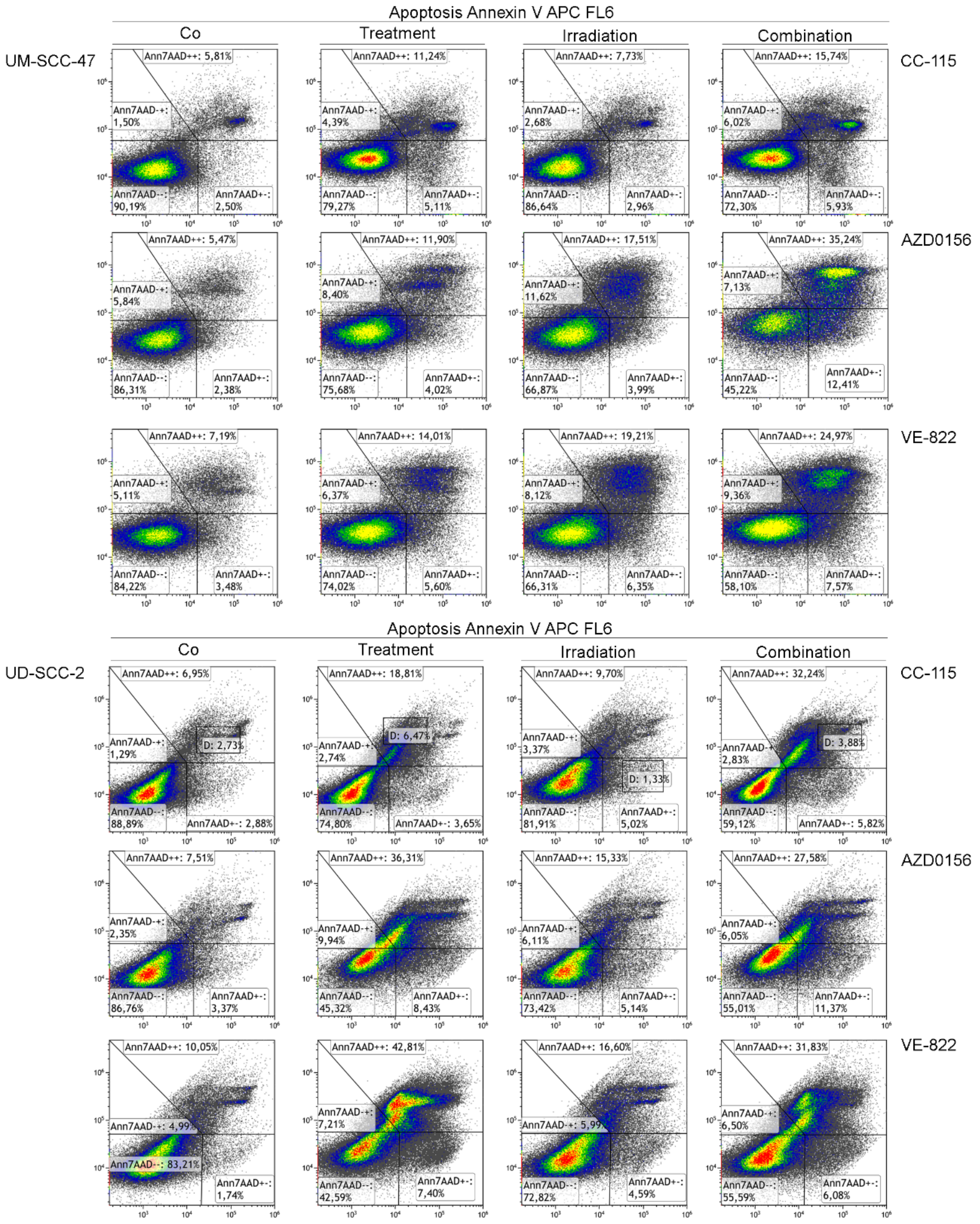

Figure S2. Representative dot plots of AnnexinV/7AAD cell death staining of the HPV-positive HSC4 and Cal33. Cells were treated with CC-115 (no inhibitor, 1 µM, 2 Gy irradiation, 1 µM + 2 Gy combination), AZD0156 (no inhibitor, 1 µM, 2 Gy irradiation, 1 µM + 2 Gy combination) or VE-822 (no inhibitor, 0.1 µM, 2 Gy irradiation, 0.1 µM + 2 Gy combination).

genes

MDPI

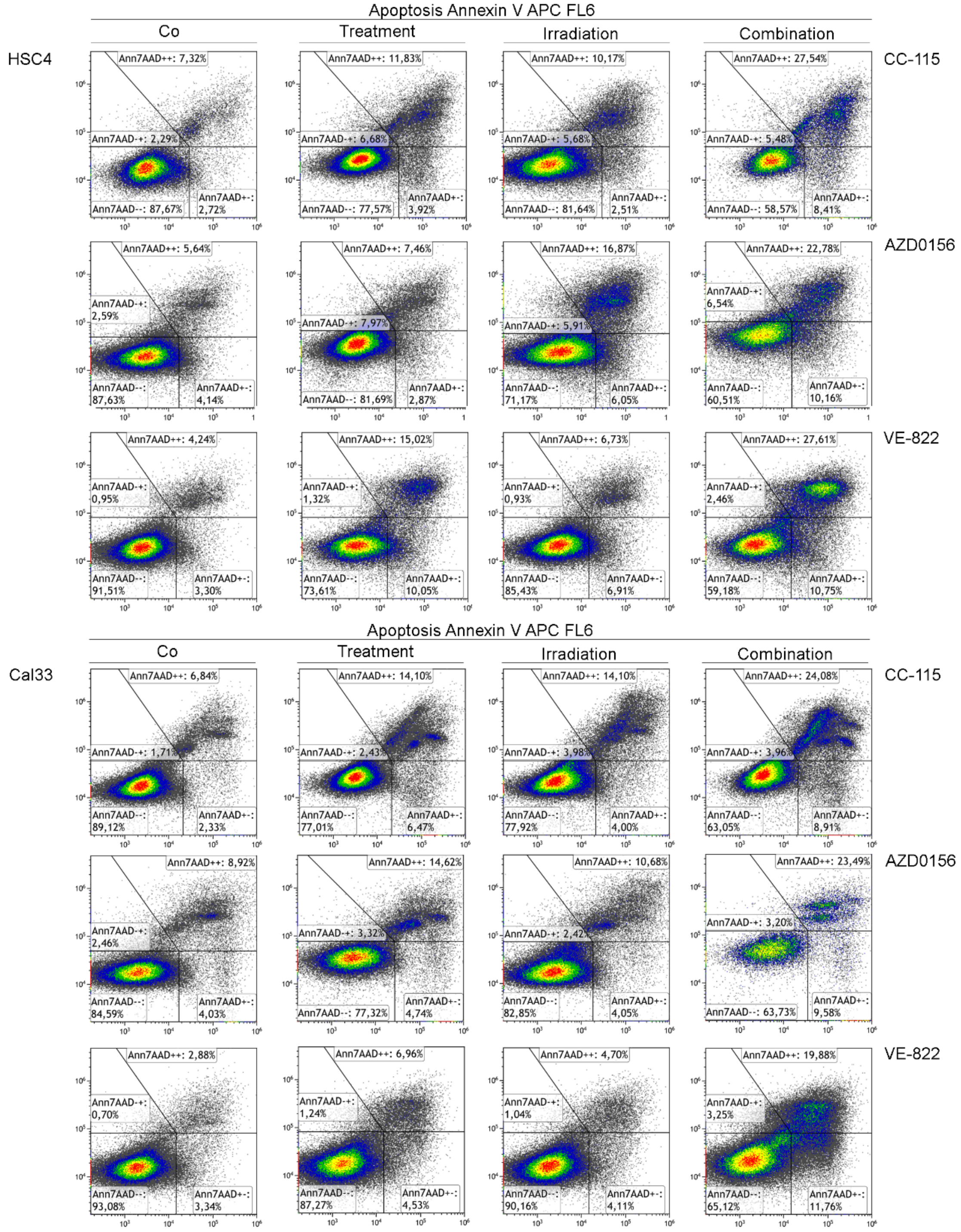

Figure S3. Representative dot plots of AnnexinV/7AAD cell death staining of the HPV-negative UM-SCC-47 and UD-SCC-2. Cells were treated with CC-115 (no inhibitor, 1 µM, 2 Gy irradiation, 1 µM + 2 Gy combination), AZD0156 (no inhibitor, 1 µM, 2 Gy irradiation, 1 µM + 2 Gy combination) or VE-822 (no inhibitor, 0.1 µM, 2 Gy irradiation, 0.1 µM + 2 Gy combination).

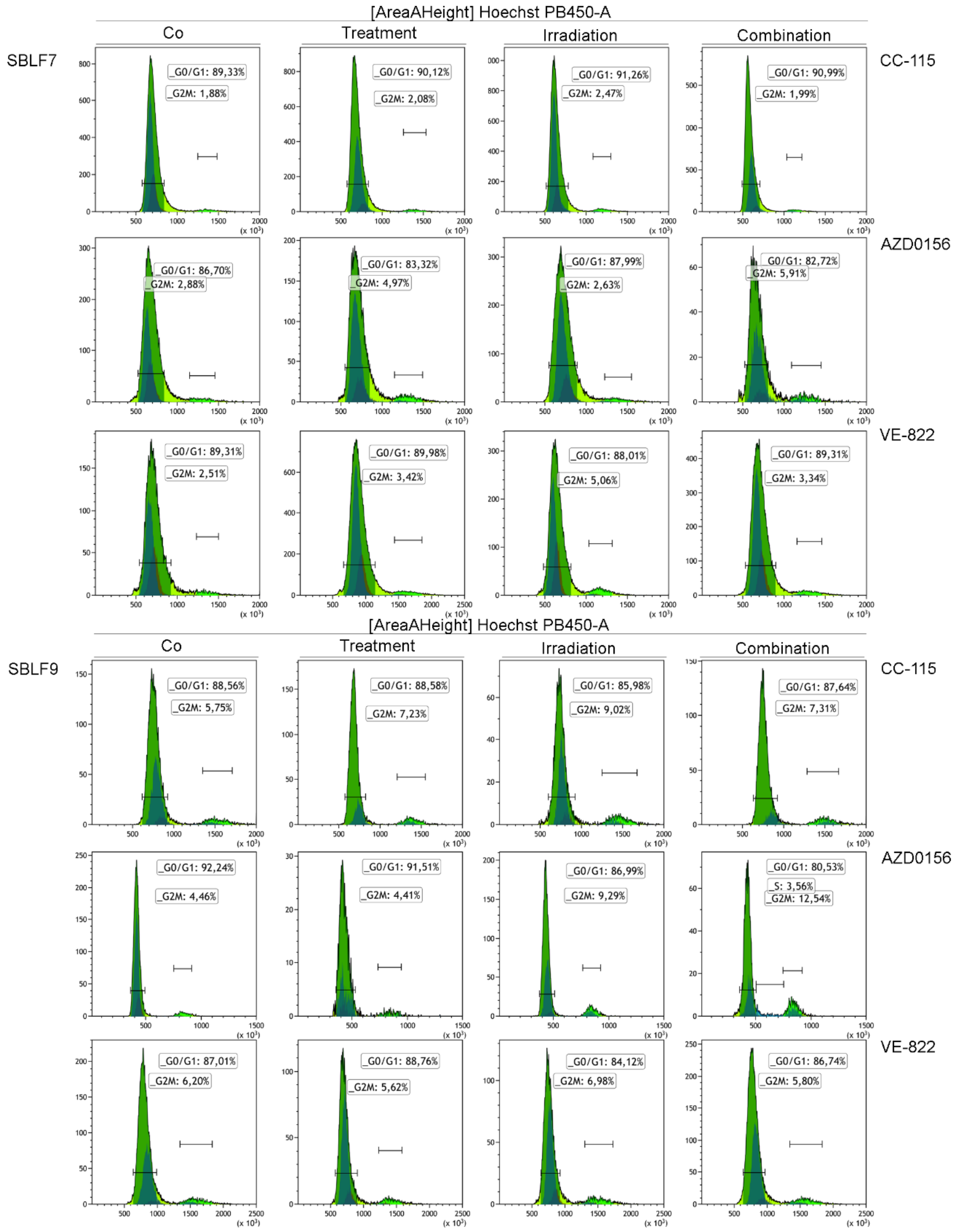

Figure S4. Representative histograms of Hoechst (DNA) cell cycle staining of the healthy fibroblasts SBLF7 and SBLF9. Cells were treated with CC-115 (no inhibitor, 1 µM, 2 Gy irradiation, 1 µM + 2 Gy combination), AZD0156 (no inhibitor, 1 µM, 2 Gy irradiation, 1 µM + 2 Gy combination) or VE-822 (no inhibitor, 0.1 µM, 2 Gy irradiation, 0.1 µM + 2 Gy combination).

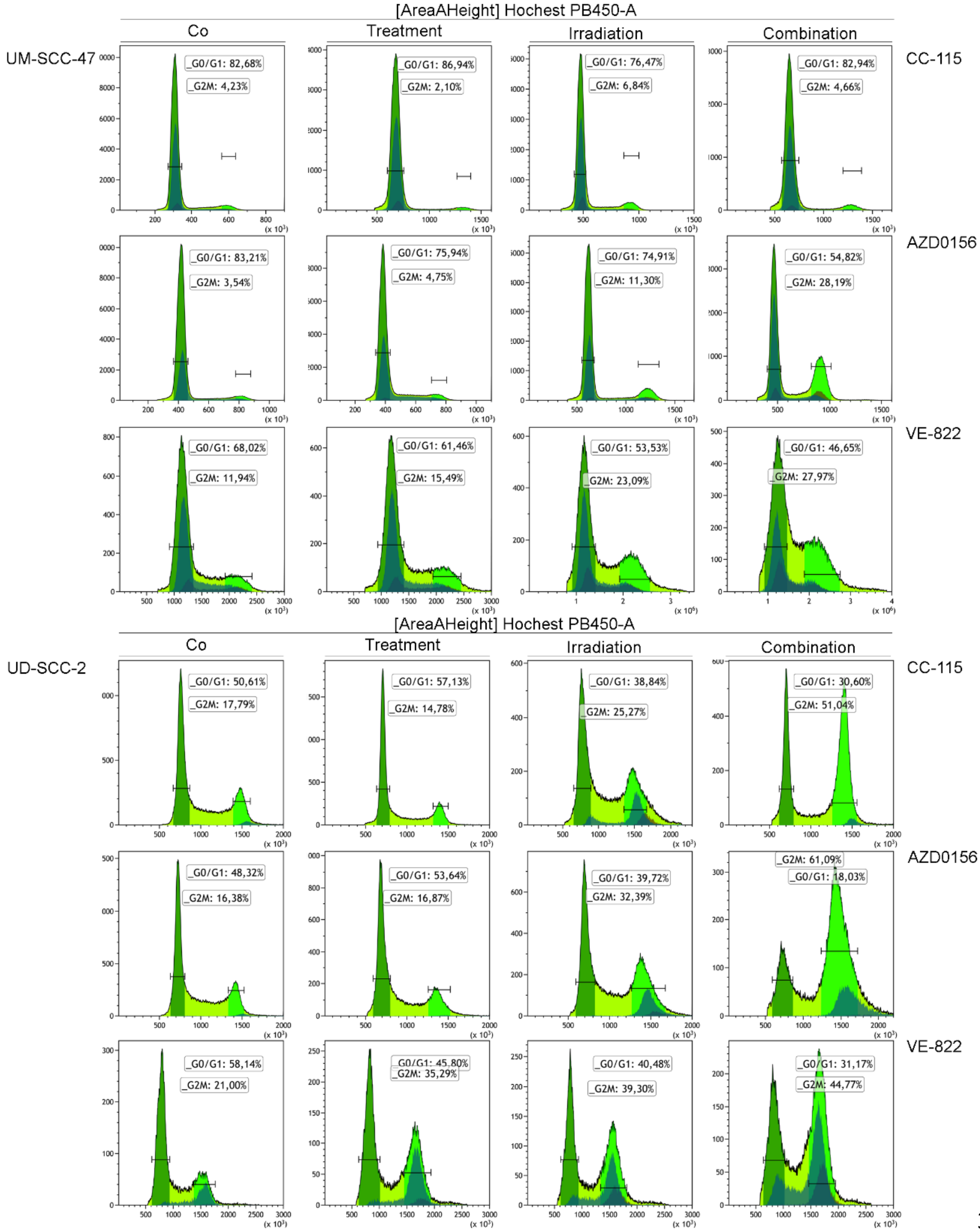

Figure S5. Representative histograms of Hoechst (DNA) cell cycle staining of the HPV-positive HSC4 and Cal33. Cells were treated with CC-115 (no inhibitor, 1 µM, 2 Gy irradiation, 1 µM + 2 Gy combination), AZD0156 (no inhibitor, 1 µM, 2 Gy irradiation, 1 µM + 2 Gy combination) or VE-822 (no inhibitor, 0.1 µM, 2 Gy irradiation, 0.1 µM + 2 Gy combination).

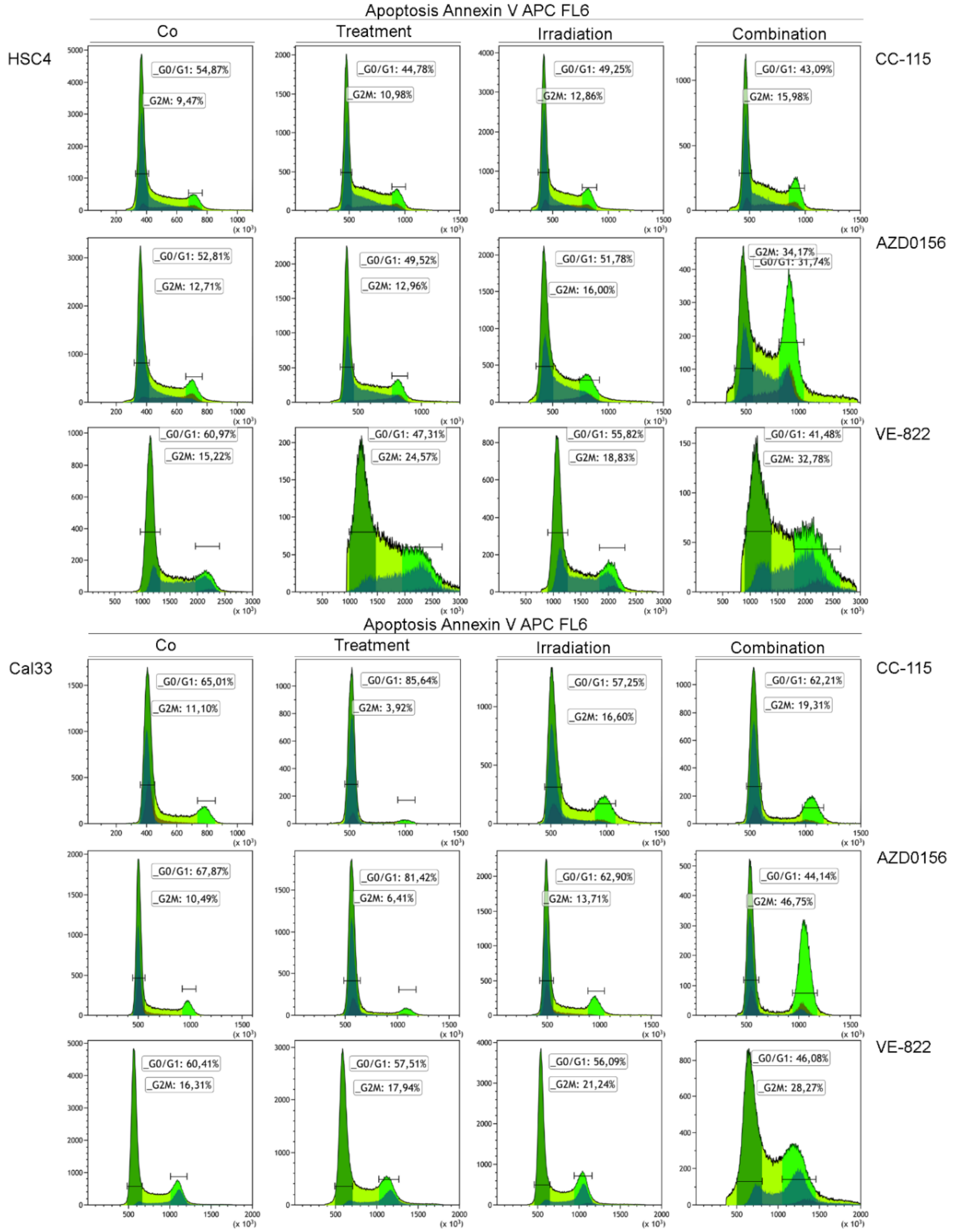

Figure S6. Representative histograms of Hoechst (DNA) cell cycle staining of the HPV-negative HSC4 and Cal33. Cells were treated with CC-115 (no inhibitor, 1 µM, 2 Gy irradiation, 1 µM + 2 Gy combination), AZD0156 (no inhibitor, 1 µM, 2 Gy irradiation, 1 µM + 2 Gy combination) or VE-822 (no inhibitor, 0.1 µM, 2 Gy irradiation, 0.1 µM + 2 Gy combination

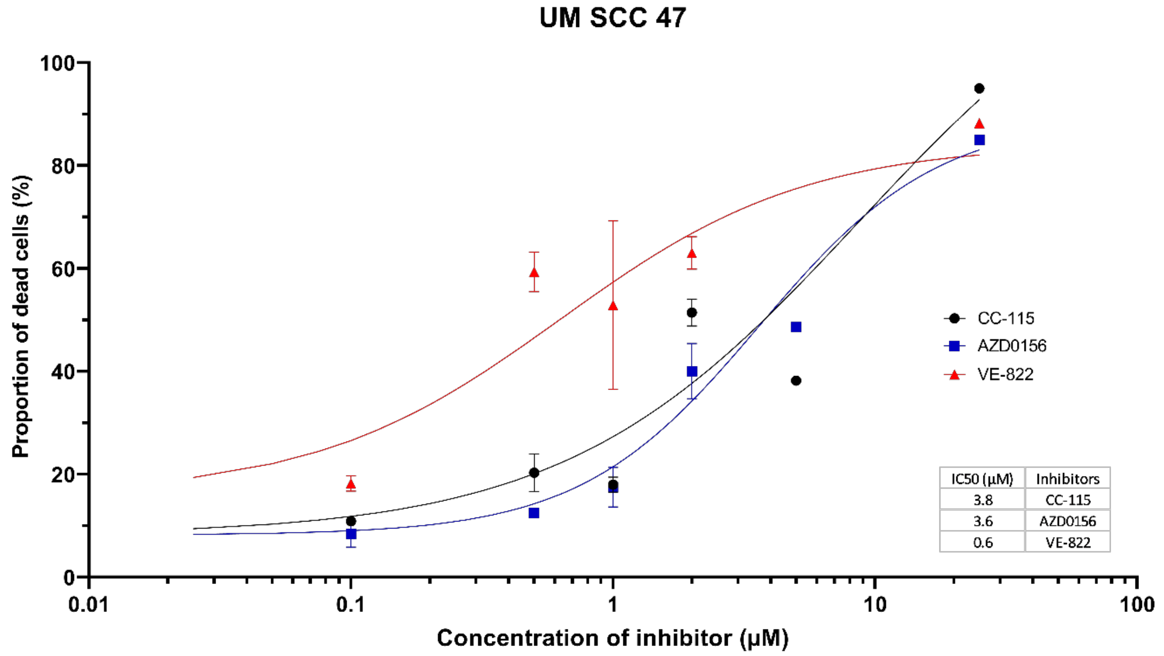

Figure S7. Dose escalation study for the induction of apoptosis or necrosis (cell death) by the inhibitors CC 115, AZD0156 and VE 822 conducted on HPV-positive cell line UM-SCC-47. Each value represents mean ± SEM.

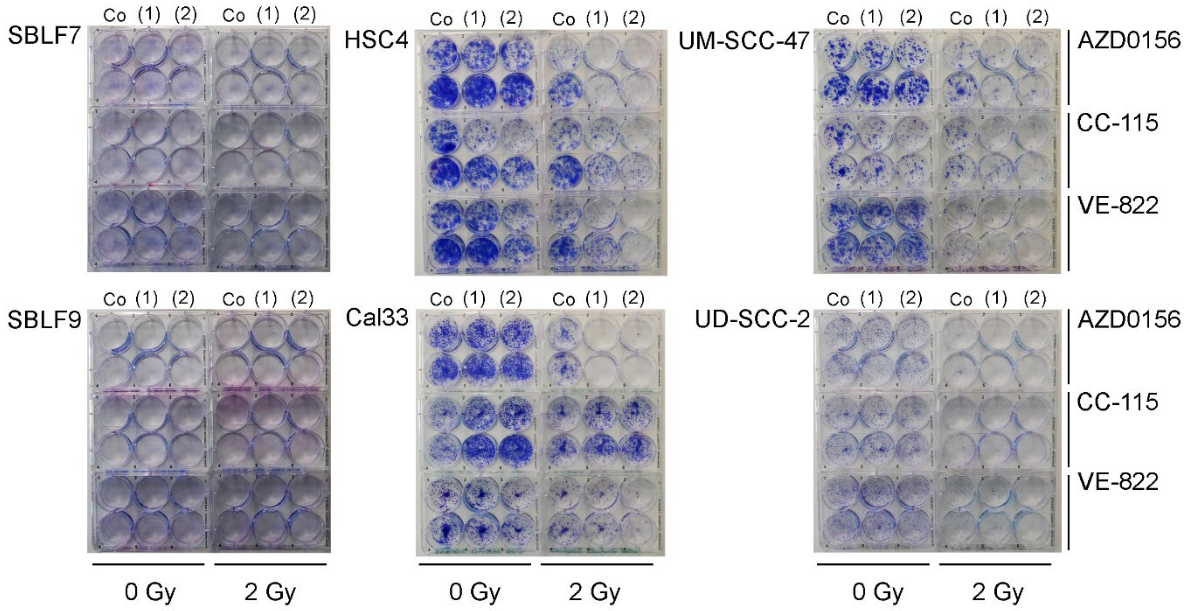

Figure S8. Colony forming assay. Representative images of all tested cell lines (healthy: SBLF7, SBLF9; HPV-neg: HSC4, Cal33; HPV-pos: UM-SCC-47, UD-SCC-2) and inhibitor (AZD0156: ATMi; CC-115: DNA-PKi, VE-822: ATRi). Cells were treated with different concentrations of inhibitors (Co: no inhibitor; (1): 5 nM AZD0156, 0.5 µM CC-115, 0.05 nM VE-822; (2): 10 nM AZD0156, 1 µM CC-115, 0.1 nM VE-822). Experiments were seeded in technical replicates (Well 1-3 and well 4-6). In case of healthy fibroblasts, typical morphology of cells leads to slightly stained colonies. Therefore, microscopical counting was done.

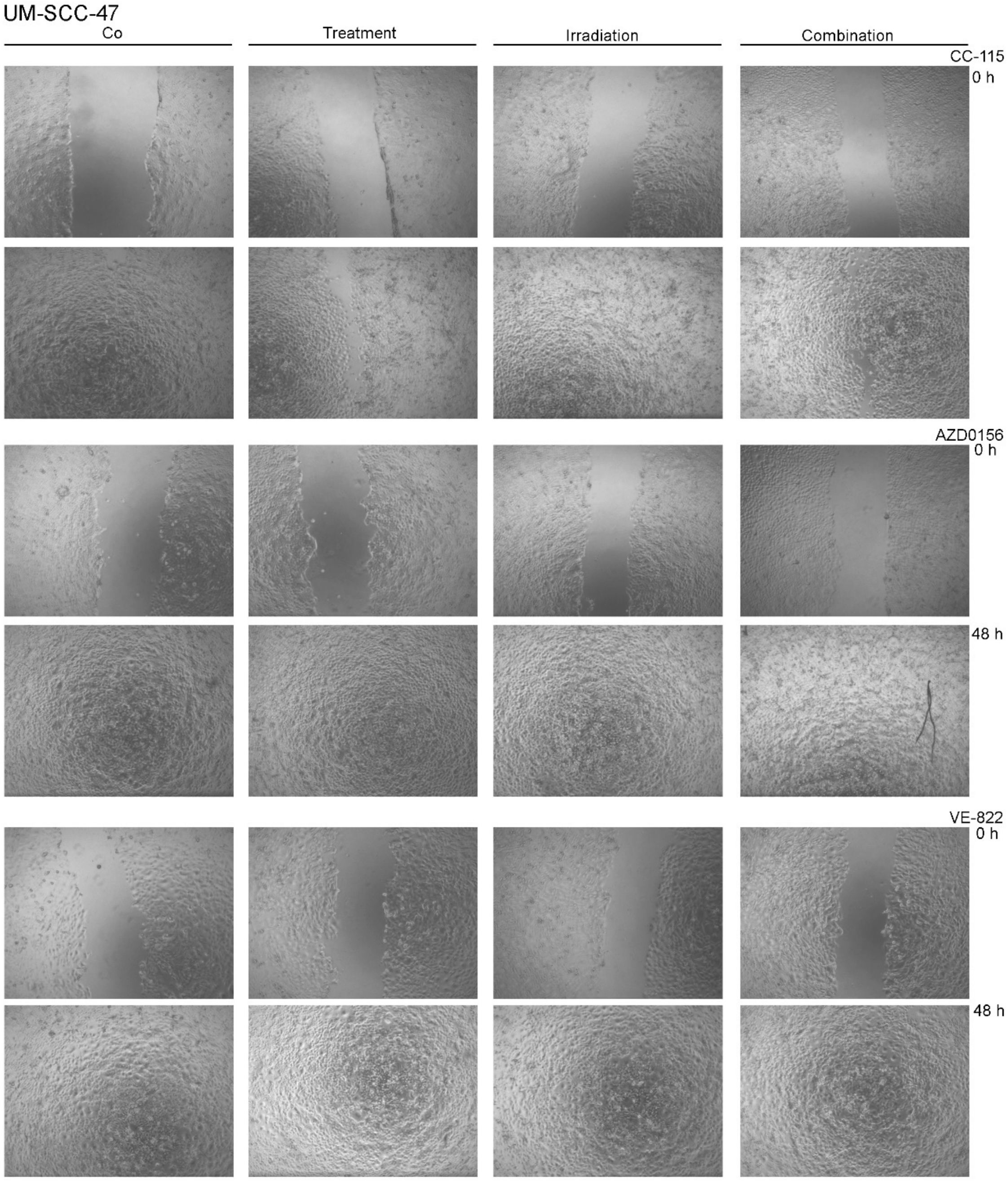

Figure S9. Representative images of wound healing assay of HPV-positive cell line UM-SCC-47 at time points 0 h and 48 h. Cell were treated either without any treatment, with inhibitor (1 µM CC-115, 1 µM AZD0156 and 0.01 µM VE-822), 2 Gy dose or a combination of inhibitor and 2 Gy.

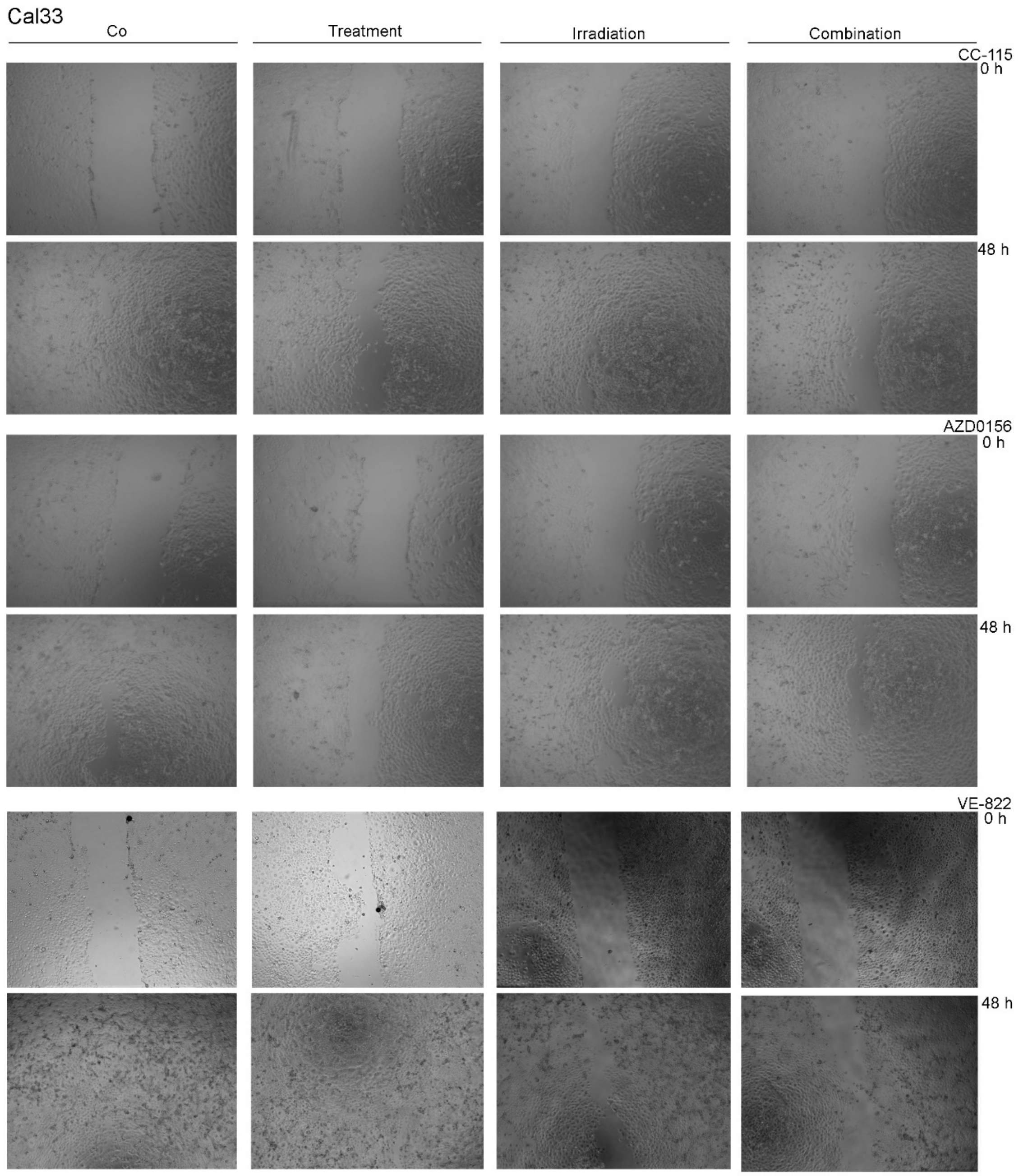

Figure S10. Representative images of wound healing assay of HPV-negative cell line Cal33 at time points 0 h and 48 h. Cells were treated either without any treatment, with inhibitor (1 µM CC-115, 1 µM AZD0156 and 0.01 µM VE-822), 2 Gy dose or a combination of inhibitor and 2 Gy.

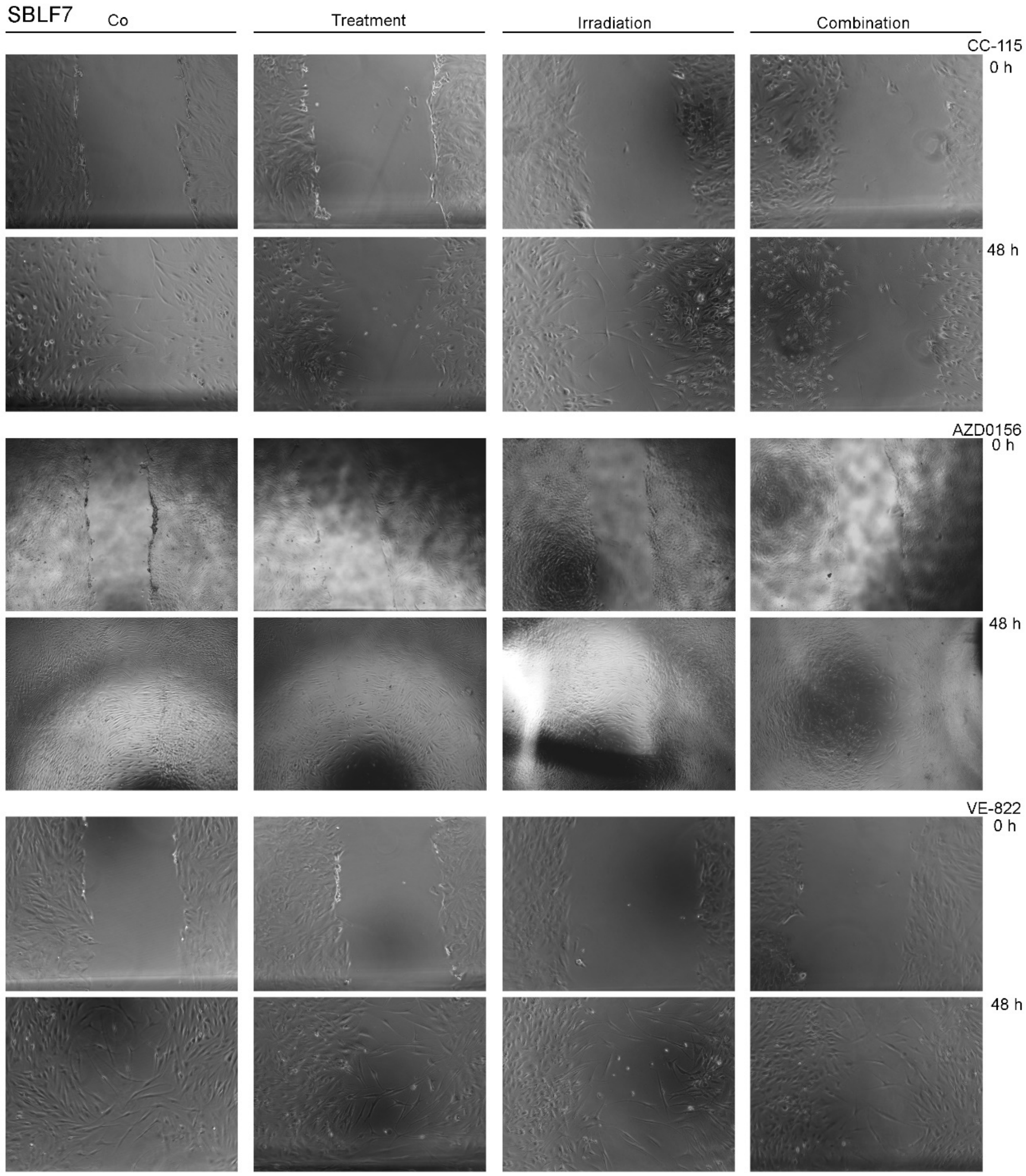

Figure S11. Representative images of wound healing assay of healthy control cell line SBLF7 at time points 0 h and 48 h. Cells were treated either without any treatment, with inhibitor (1 µM CC-115, 1 µM AZD0156 and 0.01 µM VE-822), 2 Gy dose or a combination of inhibitor and 2 Gy.
